# COVID-19 and Systemic Lupus Erythematosus: Focus on Immune Response and Therapeutics

**DOI:** 10.3389/fimmu.2020.589474

**Published:** 2020-10-30

**Authors:** Allison P. Spihlman, Nirupa Gadi, Samantha C. Wu, Vaishali R. Moulton

**Affiliations:** ^1^ Division of Rheumatology and Clinical Immunology, Department of Medicine, Beth Israel Deaconess Medical Center, Harvard Medical School, Boston, MA, United States; ^2^ Boston University School of Medicine, Boston, MA, United States

**Keywords:** COVID-19, systemic lupus erythematosus, immune response, therapeutics, coronavirus infection

## Abstract

The SARS-CoV-2 novel coronavirus has caused the COVID-19 pandemic with over 35 million cases and over a million deaths worldwide as of early October 2020. The populations most affected are the elderly and especially those with underlying comorbidities. In terms of race and ethnicity, black and hispanic populations are affected at disproportionately higher rates. Individuals with underlying conditions that cause an immune-compromised state are considered vulnerable to this infection. The immune response is an important determinant in viral infections including coronaviruses, not only in the antiviral defense but also in the disease progression, severity, and clinical outcomes of COVID-19. Systemic lupus erythematosus is a chronic autoimmune disease which also disproportionately afflicts black and hispanic populations. In lupus patients, an aberrant immune response is characterized by the presence of circulating autoantibodies, lymphopenia, aberrant T cells, and proinflammatory cytokines along with defective regulatory mechanisms, leading to immune-mediated damage to tissues. Lupus patients are often treated with immune-suppressants and therefore are immune-compromised and more susceptible to infections and may be vulnerable to coronavirus infection. While the anti-viral immune response is important to protect from coronavirus infection, an uncontrolled proinflammatory cytokine response can lead to cytokine storm which causes damage to the lungs and other organs, causing significant morbidity and mortality. Better understanding of the underlying immune response and therapeutic strategies in lupus and COVID-19 is important to guide management of this deadly infectious disease in the context of lupus and vice-versa.

## Introduction

COVID-19 caused by the novel coronavirus severe acute respiratory syndrome (SARS) coronavirus-2 (CoV-2) is a highly contagious infection with high morbidity and mortality (1). The SARS-CoV-2 is a single stranded enveloped RNA virus with the spike glycoprotein which can bind to the angiotensin converting enzyme 2 (ACE2) receptor to enter host cells (2). ACE2 is abundantly found on the type II pneumocytes of the lung alveolar epithelium and therefore the lungs are a major site of infection. However, ACE2 is highly expressed in the gastrointestinal tract, vascular endothelium and in other tissues. Acute respiratory illness spreads through inhalation of the virus leading to respiratory signs and symptoms which commonly include cough, shortness of breath, and fever but can also include gastrointestinal symptoms and neurological symptoms such as loss of taste or smell. While some patients are asymptomatic, and some may have mild illness, others with severe cases need hospitalization, intensive care and assisted mechanical ventilation and many suffer from respiratory complications which can be fatal. Accordingly, the disease course may range from a few days or weeks with full recovery, to several months with residual tissue damage thereafter. The immune response to viral infections including the novel coronavirus is two-fold—an appropriate early antiviral defense response and a subsequent inflammatory repair response. However, inadequate or delayed antiviral responses and/or an uncontrolled inflammatory response leading to cytokine storm can lead to the inability to clear virus and cause host organ tissue damage leading to worse disease outcomes. A number of abnormalities of the immune/inflammatory response have been observed in coronavirus infections (3–6). While high white blood cell (WBC) and neutrophil counts have been observed, low lymphocyte counts (lymphopenia) is a prominent finding reported in a majority of cases, and cytokine storm with elevated proinflammatory cytokine levels can precipitate complications and worse outcomes. While there is an antibody response with neutralizing antibodies against epitopes of the spike protein of the coronavirus, some reports suggest the short-lived nature of these antibodies (7).

The populations with an overwhelming preponderance of cases and deaths are the elderly >60 years of age, and of black and hispanic race/ethnicities. In most countries, higher cases and death rates are observed in men than women. In addition, those with underlying comorbidities are at a higher risk and suffer worse disease outcomes. While the most common underlying comorbidities are reported to be obesity, hypertension, diabetes, underlying cardiovascular and lung disease, people with immune-mediated diseases such as autoimmune disease, and those on immune-suppresants or immune-modulating drugs may also be more vulnerable to this infection.

**Figure 1:**
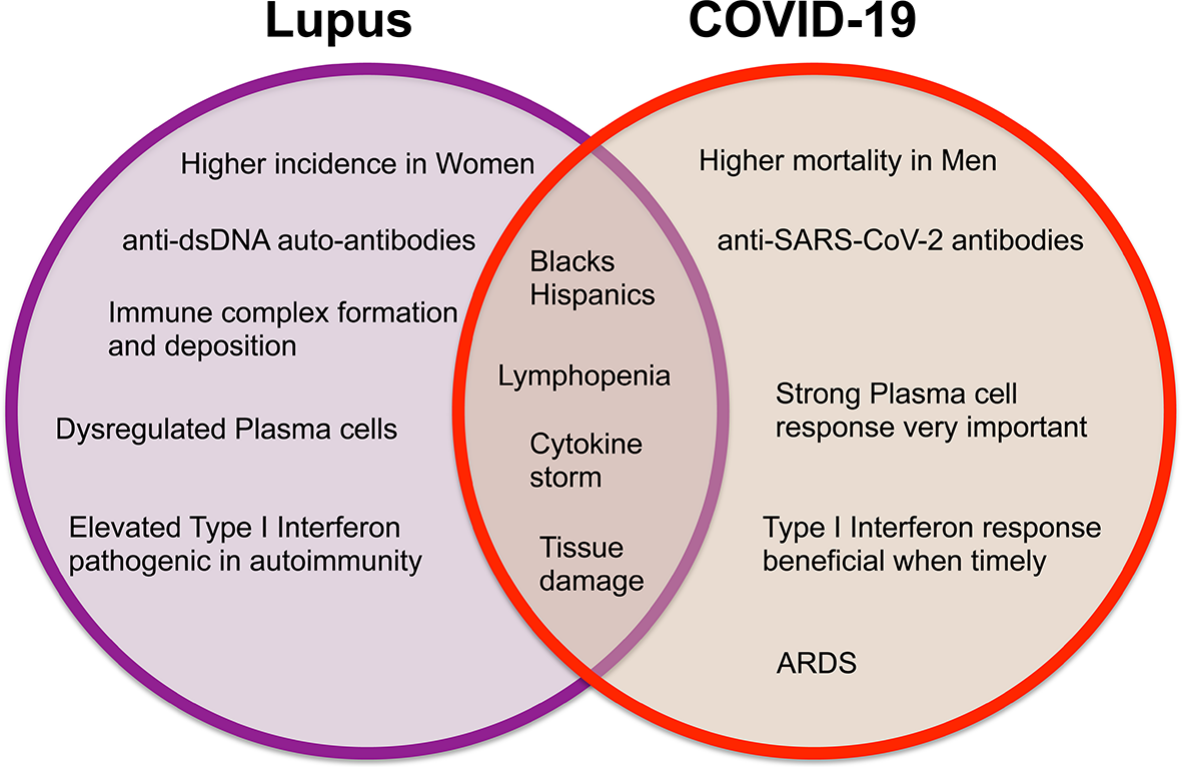
Venn diagram displaying the differences and similarities between incidence, clinical findings, and immune responses in systemic lupus erythematosus and COVID-19.

**Figure 2:**
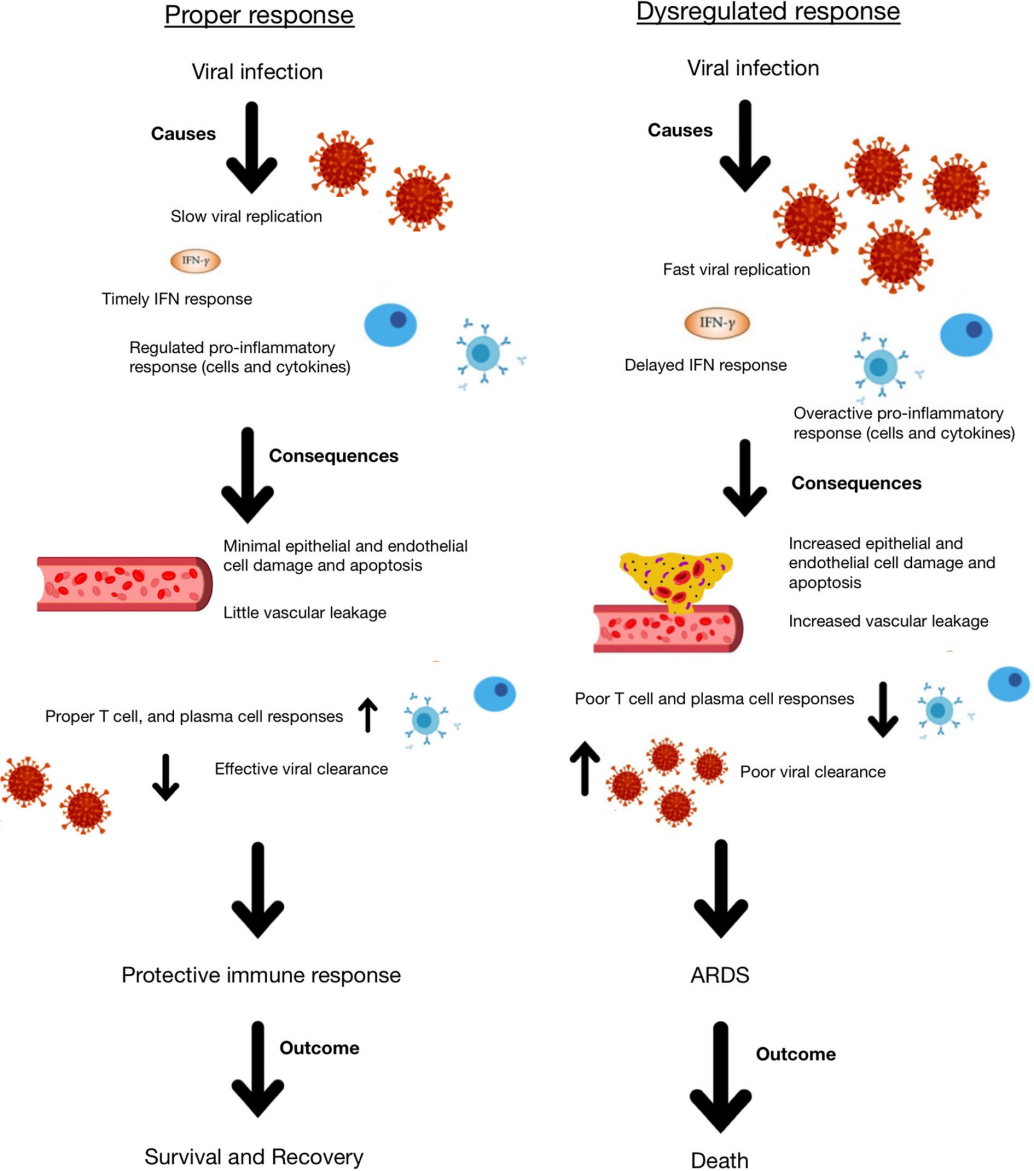
Schematic representation of appropriate and dysregulated host immune responses to coronavirus infection and the consequences of each.

Systemic lupus erythematosus (SLE) is a chronic painful autoimmune disease which afflicts predominantly women and is among the leading causes of death in young women (8). Complex interactions of genetics, hormones and environmental factors lead to loss of self-tolerance with aberrant immune responses of the innate and adaptive immune systems. Abnormal levels of innate inflammatory cytokines, autoantibodies produced by B cells and aberrant proinflammatory T cell responses lead to inflammation and tissue damage. While skin disease and joint inflammation are common, inflammation may affect virtually any organ leading to a myriad of clinical manifestations and organ damage. There is no cure for lupus and treatment with corticosteroids are often necessary to control disease activity. In addition, patients are frequently treated with immunosuppressive agents and cytotoxic drugs to control abnormal immune responses and tend to be immunocompromised and more susceptible to infections. Therefore, people with SLE are considered a vulnerable population for coronavirus infections and COVID-19. Here we discuss COVID-19 in the context of lupus, focusing on features of the immune response (**Figures 1**, **2**) including lymphopenia, cytokine storm, antibody response, T cell response, and therapeutic management strategies.

## Autoimmune Disease, SLE, and COVID-19

Several autoimmune rheumatic diseases such as systemic lupus erythematosus (SLE) result from immune-mediated inflammation and tissue damage caused by immune system dysregulation. As a result, patients with autoimmune rheumatic disease have been identified as a vulnerable population at risk for severe COVID-19 illness. Although a strong antiviral immune response is needed for viral clearance, hyperactive immunity has been linked to cytokine storm and tissue damage in COVID-19 patients (9). Both SLE and COVID-19 have been shown to manifest multi-organ complications of interstitial pneumonia, cytopenia, arthralgia, myocarditis, and hemophagocytic lymphohistiocytosis (10). Due to similarities in disease characteristics, treatment of patients with SLE may provide insights into development of therapeutic options for COVID-19.

The mainstay of treatment for SLE involves corticosteroids and other immunosuppressive therapies that may cause further vulnerability to COVID-19 infection (9). Paradoxically, immunosuppressants have been investigated as a means of dampening inflammation and reducing likelihood of acute respiratory distress syndrome (ARDS) in patients already infected with coronavirus (11). The anti-rheumatic drugs, hydroxychloroquine and baricitinib, have also been identified as possible targets for COVID-19 treatment due to their proposed antiviral effects (9). However, despite initial buzz about the use of hydroxychloroquine in COVID-19 treatment, current research suggests no benefit when compared to placebo or standard care (12, 13). Prospective studies of baricitinib therapy in COVID-19 patients have yielded more success, but are limited by poor experimental design, small sample size, and lack of results reproducibility (14). Possible adverse effects associated with baricitinib, such as secondary infection and elevated creatine kinase, have also been identified (15). Additional clinical trials are needed to elucidate the risks and benefits of these medications.

Diabetes, respiratory disease, and cardiovascular disease have been linked to more complex care and higher mortality rates in COVID-19 patients (9). However, the correlation between rheumatic disease and COVID-19 is less well-studied. Barriers to research include the relatively few individuals with rheumatic disease which limits study sample sizes. As a result, more evidence is needed to definitively determine the risk to individuals with rheumatic disease.

Current research primarily focuses on hospitalized patients which introduces bias towards those with severe COVID-19 illness. Literature suggests little if no correlation between rheumatic disease and COVID-19 disease severity in hospitalized patients (9, 16, 17). One case-control study of patients analyzed differences in disease course between patients with musculoskeletal or rheumatic diseases (cases) and those without musculoskeletal or rheumatic disease (controls) (16). In patients with severe symptoms requiring hospital stay, no significant differences in duration of symptoms, length of hospital stay, chest X-ray findings, or mortality rate were observed between cases and controls. As a result, there was insufficient evidence to support a link between musculoskeletal or rheumatic disease and COVID-19 disease course in hospitalized patients. However, due to the nature of the study, it was not possible to determine whether patients with musculoskeletal or rheumatic disease were more likely to develop pneumonia and require hospitalization. Another retrospective study reported that approximately half of reported cases of patients with both rheumatic disease and COVID-19 did not require hospitalization (17). This proportion, which included comprehensive patient data from 40 countries, appeared to be higher than that observed in the general population. However, researchers acknowledged potential bias due to data collection methods, which limited the ability to determine whether this discrepancy was statistically significant. A third study found that patients with rheumatic disease controlled with immunosuppressive medication tested positive for COVID-19 at higher rates than their family members without rheumatic disease (63 versus 34%) (9). Overall, the rate of COVID-19 in subjects with autoimmune rheumatic disease was 0.43%. Because the study selected subjects based on the criteria of having rheumatic disease or living with a family member with rheumatic disease, no conclusions can be drawn about the rate of COVID-19 infection in the general population. Though the impact of hydroxychloroquine on susceptibility of coronavirus infection remains uncertain, SLE was identified as a possible confounder in this study, as patients with SLE were more likely to take hydroxychloroquine than patients with other rheumatic diseases.

Further evidence indicates that patients with SLE test positive for COVID-19 at lower rates than individuals with other autoimmune diseases such as Sjögren’s syndrome (SS) and systemic sclerosis (SSc) (18). This is contradictory to what might be expected, as SLE is frequently treated with corticosteroids and other forms of immunosuppression which could increase vulnerability in early stages of COVID-19 infection. However, administration of low-dose steroids has also been linked to lower mortality in critically ill COVID-19 patients, which suggests that steroids may have variable effects dependent upon dosage and disease severity, further complicating understanding of how COVID-19 affects patients with SLE (19). Additionally, differences in prevalence between SLE and other autoimmune or immune-mediated disease subsets raise the possibility that anti-rheumatic therapies, such as hydroxychloroquine, might be effective in the prevention or treatment of COVID-19. Unfortunately, studies of HCQ as a preventive agent or a treatment have been disappointing (12, 13).

While more research is needed to reach a consensus on the relative risk of severe COVID-19 in individuals with SLE, evidence of abnormal clotting in some COVID-19 cases may implicate additional considerations for clinical care of COVID-19 patients who have SLE. Manifestations such as pulmonary embolism and the “covid toe” rash, stemming from thrombosis formation in the microvasculature of the feet, suggest that SARS-CoV-2 may induce a hypercoagulable state (20). In addition, prolonged activated partial-thromboplastin time (aPTT) has been observed in COVID-19 patients at higher rates than other patients, suggesting that COVID-19 may cause or exacerbate blood clotting abnormalities (21). Lupus anticoagulant (LA), a type of autoantibody which can cause hypercoagulability, was identified as the primary cause of prolonged aPTT in 91% of patients with aberrant results. These findings were replicated in a report that found 45% of severe COVID-19 patients and 87.7% of ICU patients with an abnormal aPTT also had positive LA results (22). While LA is not diagnostic of SLE, antiphospholipid antibodies, including LA, are found in approximately 50% of SLE patients and SLE is considered a risk factor for both venous and arterial thrombosis (23). Although studies have not yet confirmed an elevated risk of thrombosis among the subset of COVID-19 patients with SLE, abnormal clotting observed in both COVID-19 and SLE suggests that patients who possess both risk factors may require additional monitoring for thrombosis.

## Demographics Related Factors in SLE and COVID-19

Studies of demographic factors including ethnicity and sex have suggested that specific populations may be predisposed to more severe SLE and COVID-19 disease presentation (24). Men experience greater hospitalization and mortality rates due to COVID-19 while women experience greater incidence rates of SLE. This finding is consistent with the observation that men are biased towards infections, while women are biased towards autoimmune disorders. It has been postulated that hormonal factors which heighten ability to clear infection may also increase likelihood of autoimmune disease in women (24). Additional contributions from genetic components and microbiota may cause women to produce more pro-inflammatory cytokines which, in turn, may lead to a more aggressive immune response (24). While the process by which race and ethnicity influence risk is less clear, evidence suggests that SLE and COVID-19 disproportionately affect racial and ethnic minorities and result in worse outcomes in people of color.

Women typically mount a greater innate and adaptive immune response to infections and vaccine immune challenges (25). While a robust immune response is beneficial to fighting infection, hyperactive immunity may predispose women to autoimmune diseases such as SLE. SLE is far more common among women with a female to male ratio of 9:1. The sex-based bias in autoimmune disorders is relevant to the discussion of COVID-19 because biological factors which protect men from autoimmunity may cause worse clinical outcomes in men. This is evidenced by the more than doubled COVID-19 mortality rate observed in men compared to women, despite sometimes similar prevalence across gender (26). Studies indicate a possible genetic basis to this disparity, as many immune-related genes are X chromosome-linked (24). Typically, the silencing of one copy of the X chromosome occurs in women to ensure similar gene dosage between sexes. However, some genes may fail to undergo silencing and thus escape inactivation, producing biallelic expression of gene product (24). Biallelic expression of immune-related genes has been shown to increase T- and B-cell activation, which may both predispose women to SLE and provide protective advantages in the COVID-19 immune response.

A second explanation for the sex bias observed in both COVID-19 and SLE suggests that sex hormones may promote inflammation in women. Estrogen produces immunoactivating effects, while androgens such as testosterone yield immunosuppressive effects (25). Estrogen has been linked to increased activation of CD4 T cells and higher expression of pro-inflammatory cytokines IL-1β and IFN-γ, which may allow women to more effectively combat viral infections including COVID-19. A proposed mechanism of action suggests that estrogen may promote diversity of the microbiome, causing upregulation of certain cytokines (24). Conversely, testosterone dampens expression of pro-inflammatory cytokines while upregulating expression of anti-inflammatory cytokine IL-10, leading to a less aggressive immune response (24). Therefore, increased expression of pro-inflammatory cytokines in women which promote immune dysregulation in autoimmune disorders may simultaneously promote antiviral activity against coronaviruses.

Knowledge of disparities in disease outcome can aid clinicians and policymakers in understanding how to best treat and support patients during the COVID-19 pandemic. Emerging evidence indicates a troubling pattern of inequities in COVID-19 case rate and outcome among various populations. For instance, racial/ethnic minority communities are at increased risk of COVID-19 infection and generally suffer worse outcomes after being infected. This trend is reflected in patients with SLE. In both the United States and United Kingdom, data indicates that ethnic minorities are more susceptible to infection and mortality from COVID-19. US counties with majority black residents showed three times the rate of COVID-19 cases and six times the rate of deaths as counties with majority white residents (27). A study conducted in the UK similarly found that mortality rates in COVID-19 patients were twice as high in Bangladeshi communities and 10–50% higher in other ethnic minority communities when compared to the white British population (28).

SLE is also more prevalent in black and hispanic individuals compared to white individuals (29). In the UK, prevalence of SLE was 5–9 times greater in the Afro-Caribbean group and 2–2.4 times greater in the South Asian group than the white group. A study comparing American Indian and Alaska Native populations with black Americans found similar rates of SLE in both populations, which were higher than that observed in the white population (29). In addition, ethnic minorities may experience renal complications at higher rates, as the renal disease incidence rate for black and hispanic patients is 68.9 and 60.6% respectively compared to only 29.1% in white patients. Studies indicate similarly elevated rates of lupus nephritis in South Asian and East Asian populations compared to white populations (29).

The explanation for the increased disease risk experienced by ethnic minorities is likely multifactorial, involving both biological aspects and systemic issues which reflect pre-existing health and socioeconomic disparities in communities of color (30). Several biological models for greater disease burden in ethnic minorities have been proposed. SLE has been shown to be highly heritable, suggesting possible genetic predispositions which place ethnic minorities at higher risk (31). Higher prevalence of cardiovascular risk factors in ethnic minorities, potentially due to variable expression of ACE2, also poses additional risk of acute kidney and cardiac failure from COVID-19 (27). While more evidence is required to determine a genetic cause for this bias, preliminary data indicates that some black Americans may carry a shorter CAG repeat polymorphism in the androgen receptor gene (32). Short CAG repeat polymorphisms have been linked to increased coronavirus uptake by the ACE2 receptor and worsened COVID-19 symptoms (32).

Social and economic influences, such as those which limit healthcare access in minority communities, must also be considered. For example, lower income and education attainment are associated with higher rates of preventable disease, reduced access to health services, and shorter life expectancy. As a result, systemic inequalities which disadvantage ethnic minorities in both financial and healthcare spheres may also be reflected in trends in COVID-19 data. Patterns in employment have found that people of color are more likely to be employed in frontline jobs limiting ability to adhere to social distancing measures and increasing likelihood of disease exposure (30). While further analysis of each of these factors is needed, the impact of a pandemic which disproportionately affects already vulnerable populations must be acknowledged and addressed.

## Lymphopenia

Lymphopenia, or a decreased lymphocyte count is one of the most common features in patients with SLE with a predominant effect on T cells (33). Although it is not entirely known what causes lymphopenia in SLE patients, there are many different pathophysiological theories. The first is the presence of lymphocytotoxic antibodies. Many SLE patients show higher levels of IgM and/or IgG autoantibodies, which exhibit cytotoxicity *via* the classical complement pathway and antibody-dependent cell cytotoxicity, respectively. Some of the recognized antigens are CD4, CD45, MHCI/II, glycophospholipids, and ribosomal P protein. The majority of these are present on T cells, which may explain why T cells are more affected than B cells (33). The second theory is excess apoptosis, which may be due to a decrease in glutathione in lymphocytes. Glutathione is a powerful antioxidant that is naturally occurring in many somatic cells. In the absence of this molecule, cells exhibit an increase in reactive oxygen species and resulting apoptosis. Additionally, increased apoptosis may be due to hyper-expression of Fas on naive and memory T cells. Fas is a receptor that induces the extrinsic pathway of apoptosis. Another theory is increased susceptibility to complement-mediated cytolysis. Complement proteins are naturally occurring in the body and work to destroy pathogens or infected cells. Because of their hyperreactivity, most cells on the body have multiple complement regulatory receptor proteins on their surfaces in order to prevent complement activation on healthy cells. T cells in many SLE patients exhibit decreased numbers of these receptors, specifically CD55, CD59, and CD46. A final theory is decreased lymphocyte production (lymphopoiesis) and subsequent sequestration following production. Some SLE patients show lower numbers of CD34+ hematopoietic progenitors, leading to lower numbers of lymphocytes. Additionally, IFN-γ seems to have a negative role on stem cell production because it limits self-renewal of hematopoietic stem cells by inducing hyper-expression of transcription factor PU.1 which blocks B lymphopoiesis and blocks Pro-T cell stages in the thymus. Following production of lymphocytes, there seems to be a sequestration of these cells in secondary lymphoid organs and inflammatory sites rather than active circulation in the periphery.

Whatever the cause, lymphopenia is an important factor often associated with SLE patient susceptibility to bacterial and viral infections (33–35). Interestingly, many COVID-19 patients without SLE exhibit lymphopenia (36). 35–75% (study-dependent) of patients who have COVID-19 develop lymphopenia, defined as a lymphocyte count of <1.5 x 10^9/L (37). Additionally, this clinical finding is a more common feature in patients who died from COVID-19 (36). Furthermore, there seems to be a geographical difference in lymphopenia rates. More patients in Italy show lymphopenia when compared to places like Singapore and the Zhejiang Province. There are many reasons why this may happen, but one theory is that the viral genome has mutated and is sparking different immune responses as a result of those mutations (36). Overall, a decrease in the number of B and T cells may lead to a worse patient outcome if their ability to fight off the virus is diminished. A rapid blood test of lymphocyte count in infected patients may be useful in determining cases that are more severe, allowing physicians to respond accordingly (37). It is important to keep in mind the increased risk that COVID-19 poses to SLE patients.

## Cytokine Storm

Cytokine storm occurs when there is an excessive and uncontrolled release of pro-inflammatory cytokines and can be associated with infectious or non-infectious diseases, and the term became popular during the avian H5N1 influenza infection (38). Normally, cytokines are a critical component of the immune response, kickstarting the innate immune system and coordinating the adaptive immune response in order to ensure the rapid destruction of a pathogen, followed by tissue repair. A cytokine storm usually begins with the innate immune response to a particular pathogen (39). Toll-like receptors (TLRs) and pattern recognition receptors (PRRs) on innate immune cells recognize pathogen-associated molecular patterns (PAMPs) on pathogens. Following this interaction, innate immune cells release cytokines such as TNF-α, IL-1β, IL-6, and IFN-γ in order to stimulate the proper proinflammatory response to a pathogen. This response is normal and critical when it tapers off as expected. In a cytokine storm, however, there is an “autoamplifying phenomenon”, which is essentially a heightened response to the pathogen. It ends up causing much more harm than good, leading to host tissue damage. The most common cytokines implicated in a cytokine storm are IL-1β, IL-6, IL-12, IL-17, TNF-α, and COX-2, and endothelial cell damage is the most common feature of cytokine storm (39).

In patients who make autoantibodies or antibodies against self, such as SLE patients, cytokine storm can be induced much more readily due to increased cell damage and a subsequent increase in damage-associated molecular patterns (DAMPs) that can activate the innate immune response (39). Additionally, it is possible to see macrophage activation syndrome (MAS) in patients with SLE (40). MAS is an acute episode of inflammation marked by activation and expansion of CD8 T lymphocytes and hemophagocytic macrophages. The pathogenesis of this disease is marked by a cytokine storm of cytokines including IL-1, IL-6, IL-18, TNF-α, and IFN-γ. IL-1, produced by macrophages, causes leukocyte and endothelial activation. IL-6 drives the acute-phase response and may amplify the inflammatory response that contributes to a cytokine storm. TNF-α may induce tissue damage. IL-18 increases IFN-γ production by natural killer (NK) cells and T cells and increases TNF-α production by macrophages. IFN-γ is a prominent activator of inflammatory cytokine release (40). Many of these cytokines overlap with the classical cytokines implicated in cytokine storm and thus have the same effects on the patient. SLE patients show DNA hypomethylation of cytokine genes, particularly Type 1 interferon genes, which causes elevated levels and leaves these individuals more susceptible to a cytokine storm and tissue damage in response to a viral infection such as COVID-19 (41).

It is believed that the most severe manifestation of COVID-19 infection—acute respiratory distress syndrome (ARDS) is caused by excessive host inflammatory response rather than by the viral infection itself (42). Correlative evidence from SARS and MERS patients suggest that hyper-inflammatory responses play a strong role in coronavirus-related ARDS pathogenesis. In response to SARS-CoV-1 infection, many patients show elevated TNF, IL-6, and IFN cytokines and elevated chemokines CCL3, CCL5, CCL2, and CXCL10. These cytokine levels were particularly high in patients with severe disease when compared to patients with uncomplicated SARS-CoV-1 infection. MERS patients show similar pathogenesis to SARS patients (42).

There are many possible reasons for the initiation of the hyperinflammatory response to coronavirus infection, but it is likely due to a combination of rapid virus replication, delayed IFN response, macrophage and neutrophil accumulation, and infection of alveolar epithelial cells (42). All of these factors may lead to increased cytokine release and potential resulting cytokine storm. As a result of cytokine storm, whether in response to a coronavirus or any other infection, patients may exhibit epithelial and endothelial apoptosis, which damages the blood:air barrier in the lungs, leading to edema and hypoxia. Furthermore, there may be a diminished T cell response as a result of the delayed but hyperactive IFN response, which leads to a dysregulated immune response to the virus. As a result of this dysregulation and increased cytokine production, the tissue homeostasis in the lungs is often disrupted, leading to increased fibrin deposition that further disrupts the blood:air barrier and causes ARDS, hypoxia and permanent damage (42). IL-6, IL-8, and IL-1β are particularly important in mediating ARDS.

When compared to other causes of ARDS, COVID-19 patients with ARDS exhibited plasma IL-6 cytokine levels far below those exhibited in other non-COVID-19 ARDS patients (43). IL-6 is the main mediator of cytokine storm, and based on a meta-analysis of studies of COVID-19 patients, it is clear that the most severe cases exhibited elevated levels of IL-6, but those levels were 10–200 times lower than those seen in non-COVID-related ARDS. This argues that the lung damage and poor outcomes seen in COVID-19 patients may not be a result of a cytokine storm as seen in SARS-CoV-1 cases, but rather likely a result of a severe viral pneumonia (43). A post-mortem study of patients with COVID-19 ARDS identified severe vascular injury that was 9 times more prevalent than that seen in influenza ARDS. Given this information, cytokine storm may be a misleading description of the cause of COVID-19 ARDS and thus the treatments that patients receive may not be the best treatment option (43). The COVACTA trial, a multi-institution clinical trial that analyzed the efficacy IL-6 receptor inhibitor Tocilizumab in the treatment of COVID-19 patients, failed to meet its primary endpoint of improved clinical status. Additionally, despite Tocilizumab-treated patients spending approximately seven less days in the hospital than control patients, there was no improvement in mortality between the two groups. These trials call the use of Tocilizumab for COVID-19 patients into question and further suggest that the cytokine storm seen in COVID-19 patients is different than the classical description of cytokine storm (44).

## Antibody Response

One marker for the diagnosis of SLE is the presence of anti-dsDNA autoantibodies (45). These are formed in many SLE patients because they respond to self dsDNA as if it is non-self and thus launch an immune response against their own tissues, leading to the formation and deposition of immune complexes throughout the body. This deposition has a damaging effect on multiple tissues throughout the body, most notably the joints and vascular and renal systems.

The etiology of the production of autoantibodies is not entirely known, but it is clear that these antibodies may be formed against true self chromatin or formed due to infection-related DNA or DNA-binding proteins through molecular mimicry (45). Most commonly, the viruses that stimulate dsDNA-specific B cells and helper CD4 T cells belong to the polyomavirus group. This group of viruses remain latent after the primary infection in an immunocompetent host, which often can lead to the stimulation of these B and T cells that will attack self rather than non-self (46). Additionally, these polyomavirus infections are linked to antibodies against T antigens and transcription factors like cAMP response-element binding protein (CREB) (45). Despite various studies, the clinical significance of infection-induced antibodies to the pathogenesis of SLE is uncertain.

The interplay of environment, epigenetic modifications, production of autoantibodies, and dysregulated immune system leaves SLE patients highly susceptible to infections, with 50% of SLE patients hospitalized with an infection during the course of their disease (34). Generally, SLE patients are more susceptible to bacterial infections such as Streptococcus pneumoniae, Escherichia coli, and Staphylococcus aureus because of the lymphopenia that disproportionately affects T cell counts over B cell counts. T cells are much more important for coordinating the correct response against a bacterial pathogen. SLE patients are also more susceptible to contracting a few viruses, the most notable being Epstein-Barr virus and cytomegalovirus (34). Some of these causes of susceptibility include breakdown of epithelial barriers due to rashes and ulcers, which allow easier access for pathogens, and impairment of immune function due to the development of autoantibodies and immune complexes, which leads to low neutrophil count and dysfunction of neutrophils, basophils, and eosinophils. In addition to a hyperactive inappropriate immune response, SLE patients also exhibit increased autoreactivity of helper and cytotoxic T cells along with dysfunctional naive, memory, and plasma B cells. The dysfunctional plasma B cells, in addition to incorrect stimulation by helper T cells, can result in hypogammaglobulinemia and increased infection risk (34). Patients are sometimes given glucocorticoids to control disease activity, but these drugs are powerful immunosuppressive drugs that further increase a patient’s risk of infection and a poor immune response.

It remains uncertain how SLE patients will respond to COVID-19 and whether they are at an increased risk to contract the infection. Although it may seem as though SLE patients are more susceptible to contracting COVID-19 because of their maladaptive immune system, it is interesting to note that there have been few cases of COVID-19 patients who also have SLE (35). The Global Rheumatology Alliance registry, as of April 1, 2020, noted 19 patients over multiple continents who have SLE and diagnosed COVID-19. A follow-up study found that individuals with rheumatic disease who took >/= 10 mg/day of Prednisone were more likely to be hospitalized, whereas individuals with rheumatic disease who took TNFα were at a decreased risk of hospitalization. Individuals with rheumatic disease using NSAIDs or anti-malarials, such as hydroxychloroquine (HCQ) saw no difference in hospitalization risk. Because many SLE patients are on medications such as glucocorticoids (Prednisone) and HCQ, it is possible that this population is at an increased risk for hospitalization, should they contract COVID-19 (17).

Although studies have noted a production of anti-SARS-CoV-2 antibodies in patients with mild, moderate, and severe COVID-19, it is uncertain the role these antibodies play in mediating the immune response to the disease (47). Because SLE patients show abnormally high levels of autoantibodies, impaired generation of antibodies against pathogens, and are frequently using DMARDs such as glucocorticoids, they may be at high risk for both contraction of COVID-19 and hospitalization should they become infected.

## T Cell Response

SLE is characterized by excessive production of autoantibodies from B cells which instigates systemic inflammation. Because of this, SLE was originally thought of as a disease mediated by abnormal B cells and plasma cells (48). In reality, aberrant T cells are equally if not more important key initiators of the observed systemic inflammation, as they stimulate proliferation, maturation, and differentiation of B cells, thereby enhancing autoantibody production and class-switching in SLE. Hyperactivation of T cells in this autoimmune disease is relevant in the context of COVID-19, as stimulation of the adaptive immune system after infection may predispose SLE patients to more severe outcomes.

In SLE, there are several phenotypic and physiological changes that are observed in T cell receptors and signaling. CD3ζ, also known as CD247, is a T cell surface glycoprotein responsible for coupling antigen recognition with downstream signal transduction. This surface protein has poor expression in patients with SLE and other inflammatory disorders, causing preferential activation of the Syk pathway for intracellular signaling. This alternative pathway confers much stronger activation of signaling molecules and calcium flux which all intensify the T cell response (48). This process leads to downstream transcription factors which upregulate production of CD40 ligand (CD40L) in patients with SLE. CD40L is a costimulatory molecule on T cells and interacts with CD40 on B cells to promote differentiation, proliferation, antibody production, and class switching in B cells (49).

A common pathway of T cell activation in SLE patients has been outlined in the previous paragraph, as many of these intermediates have been targeted as sites of dysregulation in COVID-19 disease, where T cell hyperactivation is implicated in the infamous cytokine storm. For example, CD40L enables collaboration of T cells with B cells. In patients with SLE, this ligand is upregulated by activation of the Syk pathway (48, 49). This can be problematic as CD40L has additional functions within several cell types of the vasculature, including endothelial cells, myocytes, and platelets. They have strong roles in positive feedback mechanisms of inflammation, as they are released from platelets to activate thrombosis (50, 51). Having SLE is actually an independent risk factor for development of arterial and venous thrombosis that is tied to this upregulation.

As related to COVID-19 patients, CD40L elevation is actually a marker of progression to critical illness. ICU patients with COVID-19 compared to controls have significantly higher levels of platelet and T-lymphocyte CD40L (51). This is concerning as SLE patients may already be in a hypercoagulable state, and development of pulmonary emboli (PE) and deep vein thromboses (DVT) are common complications of COVID-19 disease (10, 21). A recent study (21) found that over 90% of COVID-19 patients that had a prolonged activated partial thromboplastin time (aPTT) were at increased risk of thrombosis. These patients actually had lupus anticoagulant proteins that although function as anticoagulants *in vitro*, are strong procoagulants *in vivo*. The results of this study are clinically significant, as anticoagulation therapy should not be avoided in COVID-19 patients despite their prolonged aPTT, as lupus anticoagulants are responsible for many of these paradoxical test results (21). While not all patients with lupus anticoagulant are diagnosed with SLE, patients with SLE are much more likely to produce this antibody than the general population and should be monitored for thrombosis if they develop COVID-19.

Th17 cells are a newly discovered, differentiated subset of CD4 T lymphocytes defined by their production of the proinflammatory cytokines IL-17 and IL-22. They are crucial mediators of local inflammation, and typically attract other proinflammatory cell types such as neutrophils and Th1 cells at later stages of inflammation (52). SLE patients with active symptoms are found to have a higher proportion of Th17 cells and serum IL-17 levels compared to the healthy controls, and Th17 lymphocyte numbers are positively correlated with SLE disease activity and severity (53). In addition, autoantibody production is also mediated by IL-17 activation of peripheral blood mononuclear cells (PBMCs) from patients with lupus nephritis (54). To compound effects, IL-17 is capable of upregulating IL-6 production, creating an easily stimulated positive feedback loop between these inflammation-prone cytokines (55). For these processes, Th17 cells are the lymphocytes most implicated in autoimmunity and inflammatory disorders.

The mammalian or mechanistic target of rapamycin complex (mTORC) is a nutrient sensor within multiprotein complexes that control the cell cycle. Dysfunction in mTOR signaling is involved in many pathological states such as cancer and especially autoimmunity, as mTORC has important roles in the activation and polarization of naive T cells (48, 56, 57). In several studies, mTORC was shown to promote Th0 divergence into Th1 and Th17 lymphocytes, and *in vivo* inhibition of mTORC reduces the proportion of these cell types in the lamina propria and lymph nodes. In SLE T cells, the activity of mTORC1 is enhanced compared to T cells from healthy patients. In a human case study of a knockout for a negative regulator of mTORC1, the patient developed an unusually severe case of SLE that led to death (48).

Th17 lymphocytes have been linked to increased disease severity in a variety of coronavirus diseases including SARS, MERS, and COVID-19 (24). As mentioned, this relation is strongly connected to the hallmark IL-17 levels that are upregulated in infected hosts. However, people with SLE and other autoimmune disorders are at special risk of secondary complications due to unique T cell dysregulations. Hyperactivity of mTORC is only one of the many possible reasons behind the disproportionate complications in COVID-19 patients with lupus and other pre-existing autoimmune diseases (48).

## Therapeutic Strategies

Vasculitis, leukopenia, lymphopenia, thrombocytopenia, and several other complications that are typically associated with autoimmune diseases seem to be mirrored by the novel coronavirus COVID-19. It is for these similarities and their disease pathophysiology that there is an interesting overlap between SLE and COVID-19 therapy. This also introduces the question of how to treat COVID-19 patients with pre-existing SLE. Steroids and other immune-suppressive drugs are the mainstays of therapy for SLE patients rendering them immune-compromised and potentially vulnerable to COVID-19.

### Antimalarials

Chloroquine (CQ) and its less toxic derivative hydroxychloroquine (HCQ) have been used for years as treatment for autoimmune and inflammatory disorders, and are typically considered first- line treatment for SLE. Their mechanisms of action are through dual manipulation of antigen-presenting cell signaling—alkalinization of lysosomes and blocking of toll-like receptor (TLR) signaling (10). Their use reduces disease activity of SLE, reduces inflammatory damage, and improves patient survival (58). CQ/HCQ have also been shown to have an *in vitro* antiviral effect on SARS-CoV-2 *via* ACE-2-SARS-CoV-2 binding interactions (59), leading many to consider their use as a treatment for COVID-19. However, there is little valid scientific evidence to support their use, as studies of HCQ as a preventative agent or a treatment for COVID-19 have been disappointing (58, 60, 61). In fact, the World Health Organization (WHO) discontinued its use during its Solidarity trial (https://www.who.int/emergencies/diseases/novel-coronavirus-2019/global-research-on-novel-coronavirus-2019-ncov/solidarity-clinical-trial-for-covid-19-treatments) when it proved to make no significant improvement in the complications of COVID-19 patients (60). Other clinical trials done in China and France suggested HCQ use with azithromycin could result in a rapid decrease in viral shedding, but these studies were also faulty as they were uncontrolled and underpowered. The few pieces of conclusive evidence on CQ/HCQ in combination with azithromycin recognize their potentially dangerous impact on the electrochemical properties of the heart. To elucidate, these drugs are capable of lengthening the QT interval *via* blocking of K+ channels, precipitating cases of ventricular arrhythmia (62). Another recent randomized controlled trial has shown no clinical benefit of HCQ for COVID-19 (63). The lack of evidence-based recommendation of CQ/HCQ coupled with media speculation on its use as a COVID-19 therapy led to a worldwide shortage of these essential medications for patients suffering from SLE (58). Therefore, it is not recommended that antimalarial drugs be administered to prevent or treat COVID-19. Patients with SLE who are experiencing low availability in their prescriptions should consult a physician and consider tapering their dosage or increasing drug interval time until international drug shortages are ameliorated (58, 60).

### Glucocorticoids

Treatment for SLE may include a combination of antimalarials with a glucocorticoid such as prednisone (64). In the context of COVID-19, steroid use as a prophylaxis or treatment has been controversial through several conflicting studies. One of the most well-known clinical trials from the RECOVERY Collaborative Group found that the daily dexamethasone in hospitalized COVID-19 patients led to reduced incidence of death for those receiving invasive mechanical ventilation (19). On the other hand, reports from the COVID-19 Global Rheumatology Alliance have shown that people who use glucocorticoids such as prednisone at concentrations >10 mg/day are more likely to be hospitalized for COVID-19 (10, 17). A recent metanalysis found that use of glucocorticoids as a COVID-19 treatment may increase the risk of death in patients with coronavirus infections of a mild course (65). Because of this, most providers have suggested tapering dosage of glucocorticoids to below 10 mg/day in patients with rheumatic diseases or using a minimal possible dose to reduce risk of complications. For patients already taking corticosteroids, completely stopping use of glucocorticoid medications is strongly advised against (17).

### IL-2 Agonists

IL-2 agonists have great potential in T cell manipulation, as they have a strong ability to redirect the host immune system towards tolerance by the upregulation of Tregs (64). In several clinical trials in SLE patients (66, 67), low-dose IL-2 therapy was shown to have strong potential of expanding Treg populations (68). There is currently a clinical trial in Paris studying the use of low-dose IL2 in COVID-19 patients (https://clinicaltrials.gov/ct2/show/NCT04357444). However, to the best of the authors’ knowledge, there is still no widely available IL-2 agonist on the market for SLE or COVID-19 therapy.

### IL-6 Antagonist

IL-6 is a cytokine that is strongly correlated with inflammation and severity in SLE and COVID-19. As a therapy for rheumatic illnesses, this connection was utilized *via* development of recombinant human monoclonal antibody tocilizumab targeting the IL-6 receptor that could be used for rheumatoid arthritis, certain types of juvenile arthritis, and giant cell arteritis. Additionally in 2017, the Federal Drug Administration (FDA) approved the use of tocilizumab, a humanized anti-IL6 receptor antibody, for the treatment of cytokine release syndrome (69). Recent studies on the use of tocilizumab as a therapy for COVID-19 disease have had mixed results, although this drug has been used frequently in acute care settings as a therapy for the COVID-19 cytokine storm (64, 68).

### mTOR Antagonists

Rapamycin is the most well-known mTOR pathway inhibitor, resulting in immediate and delayed inhibition of mTORC1 and 2, respectively. In a mouse model, rapamycin was able to ameliorate lupus nephritis as well as increase levels of circulating IL-2. Rapamycin has also shown these effects in clinical trials in human subjects, although it is not commonly used to treat lupus (64, 70). There are currently clinical trials using rapamycin to protect elderly patients from COVID-19 complications, although there is still little information on the effects of rapamycin use against coronaviruses (71).

### NSAIDs

Many patients with SLE use nonsteroidal anti-inflammatory drugs (NSAIDs) to alleviate joint pain and other symptoms (17). The COVID-19 Global Rheumatology Alliance has found no increased risk of poor COVID-19 outcomes related to the use of NSAIDs (10). However, a report mentioned that NSAIDs can modulate ACE2 and aggravate COVID-19 symptoms (72). From this article, the U.S. Food & Drug Administration (FDA) advised patients to be cautious of the use of NSAIDs and advocated for use of an alternative such as ibuprofen/paracetamol (https://www.fda.gov/drugs/drug-safety-and-availability/fda-advises-patients-use-non-steroidal-anti-inflammatory-drugs-nsaids-covid-19).

### TNF Antagonists

Antibodies against TNF have been used for years as treatment for autoimmune diseases such as rheumatoid arthritis and inflammatory bowel disease. This blockage of TNF in patients with autoimmune disease leads to a rapid decrease in IL-1, IL-6, and subsequent leukocyte trafficking (73). In this regard, literature promoted by the COVID-19 Global Rheumatology Alliance suggests that patients with rheumatic illness taking anti-TNF therapy had a significantly reduced odds of hospitalization (17). TNF antagonism has been strongly advocated for in COVID-19 research development, although it has not yet been tested as a therapy in clinical trials (73).

## Conclusions

Patients with autoimmune diseases such as lupus are not only vulnerable to infections because of the aberrant immune responses inherent to the disease, but also due to the fact that they often are treated with steroids, other immune-suppressants and immune-modulator drugs. These together lead to an immune-compromised state and an increased risk for infections. Many aspects of lupus and COVID-19 are shared including some demographics of patient populations affected and aberrant immune responses while some such as gender-bias are strikingly distinct in the two diseases, with lupus predominantly afflicting women and COVID-19 with worse outcomes in men. Aberrant cellular, humoral and cytokine immune responses including lymphopenia, proinflammatory cytokines, aberrant B and T cell responses may likely influence the severity and disease outcomes of COVID-19 in patients with immune-mediated and autoimmune diseases. Better understanding of the intricacies of the immune response will be important in guiding management strategies for these patients.

## Author Contributions

VM conceptualized the review. AS, NG, SW, and VM performed literature reviews, synthesized relevant information, and wrote the manuscript. AS prepared the figures. All authors contributed to the article and approved the submitted version.

## Funding

VM is funded through an NIH NIAMS grant (R01 AR076894).

## Conflict of Interest

The authors declare that the research was conducted in the absence of any commercial or financial relationships that could be construed as a potential conflict of interest.
